# Practice Management During a Pandemic: Common Issues That Affect All of Us

**DOI:** 10.1093/asjof/ojaa017

**Published:** 2020-04-29

**Authors:** Mark L Jewell, Mary Lind Jewell, Robert Singer

**Affiliations:** 1 Clinical Professor of Plastic Surgery at Oregon Health & Science University, Portland, OR; 2 practice manager in private practice, Eugene, OR; 3 (Voluntary) Professor of Plastic Surgery, The University of California, San Diego (UCSD), La Jolla, CA

On Wednesday, March 11, 2020, the World Health Organization declared the Covid-19 coronavirus outbreak that originated in China a worldwide pandemic.^1^ The effect of this has been a worldwide crisis of many dimensions beyond contagion, sickness, and death. It has stalled the world’s economy and put business and medical practices on standstill overnight and created the largest unemployment rate in decades. Most of The Aesthetic Society members are in the private practice of aesthetic plastic surgery either as sole practitioners or as small groups. They are extremely vulnerable to a disruption where elective surgery and nonsurgical procedures are curtailed. Offices are shuttered, and staff cannot work. Since March 11, 2020, over a million individuals would be infected, almost 100,000 worldwide (as of today) would die, and the world’s economy as we know it ceased to exist.

At that point, we collectively thought that there was a great opportunity for The Aesthetic Society to connect with its members as we all were struggling with how to proceed. We decided to offer a practice management webinar series to help members deal with the effect of the pandemic on their patients, practices, their lives, and the economy. For the last 25 years at The Aesthetic Society annual meetings, we have offered a comprehensive practice management instructional course. The curriculum has evolved over the years to include topics of disruption, personnel, team building, patient communication, service recovery, and social media. Given the pandemic as a “sum of all fears” scenario for disaster, we strategized on a webinar curriculum to address important common issues that affect all of us. Kudos to The Aesthetic Society’s leadership and its Education Commission for the green light to start the webinar series. Thanks are also in order to Sientra for sponsoring the webinar series and for technical assistance in using the Zoom online webinar platform (https://zoom.com; Zoom Video Communications, Inc., San Jose, CA). For those who were not able to see the Webinar live, the content has been archived and preserved and is accessible on the www.surgery.org/private/webinars site and also available on RADAR. This webinar was the first of its kind and set the standard for member service from a major plastic surgery society. The Aesthetic Society led the way in terms of a quickly developed webinar series in response to the coronavirus disaster, setting a standard for addressing the concerns of plastic surgeons and creating clarity out of general panic.

Disasters and disruptions come in all sizes and types, yet this one was different in terms of its incredible spectrum of contagion, infection requiring hospital care, death, home confinement, and standstill of the world’s business economy. With the economic impact of no work, it becomes a liquidity crisis for businesses that can quickly progress to insolvency. We collectively envisioned and presented a different playbook for recovery than in earlier economic disruptions caused by 9/11 or banking in 2008.

First, we saw this disaster as unfolding daily right in front of us, affecting the health of our patients. Our practices were closed, we could not perform elective aesthetic surgery or cosmetic medicine, and cash flow has stopped. We felt a responsibility for our staff, who need to be paid along with our creditors and landlord.^2,3^ An opportunity existed, to connect with our members in the United States and internationally to overcome feelings of doom and to harness a connected community to address these issues. Our first webinar was developed along the line of one of Franklin Delano Roosevelt’s Fireside Chats during the Great Depression where a thoughtful conversation was developed that gave individuals the opportunity to believe in themselves and have a call to action. Times have changed from listening to the President over a depression-era vacuum tube radio to a sophisticated video webinar program; however, the message of hope, leadership, resolve, and recovery still remain key elements.

The first webinar ran over 2 hours and addressed key topics of webinar listed in Table 1. It has more registrants than could be accommodated and hundreds of submitted questions. Our webinar faculty gave individual expert perspectives and ideas that offered the attendees ways to address the disruption and start planning for recovery. Some of the salient points for practice managers are listed in Table 2.

**Table 1.** Webinar Key Topics

• Personnel
• How to remain financially liquid
• Patient communications
• Compliance with advisories regarding nonessential surgery
• Addressing previously scheduled procedures
• Telemedicine and compliance with legal issues
• Expansion of office-based surgery for recovery (ie, changing facility accreditation status)
• Practice management
• Economic survival—Payroll Protection Program and Small Business Administration Disaster loans
• Office hygiene and decontamination
• Recovery planning
• Criteria for proceeding with future elective surgery

**Table 2.** A Practice Manager’s Response to the COVID-19 Disruption

• Assemble a team of experts, your banker, attorney, and accountant
• Analyze cash flow in terms of inflow and accounts payable
• Project financial obligations throughout the calendar year (ie, rent, malpractice, and maintenance)
• Look at ways to cut costs and have a leaner practice
• Each employee has a role in the recovery plan
• Continually communicate with patients and let them know that your practice is still here for them with regularly posted messages of patient concern
• Develop a recovery business plan that will allow forgiveness of Payroll Protection Program loan
• Keep employees on the job; there’s plenty of administrative work and planning to be done that there’s never time to accomplish during normal times
• Highlight your practice’s efforts to help others during the COVID-19 pandemic

The webinar series continues to evolve with regards to the tactical response of dealing with patients seeking aesthetic plastic surgery and cosmetic medicine as we move further into and out of the pandemic. From a strategic perspective, the focus should be on recovery, reconnection with our patients, the restart of elective surgery, and personal health. Some practices individually offer office-based surgery and injectables that can be restarted ahead of other facilities when the green light is given and before hospitals and ambulatory surgery centers resume accepting nonemergency elective cosmetic procedures.^4^This represents a great opportunity for the leader within all of us to become a champion. The element of resolve and perseverance must be an inherent part of the survivor’s mentality. All of us can recall the stories of individuals who have survived the Great Depression, the Holocaust, and natural/manmade disasters and how they managed during extremely difficult times.

Medical care and elective surgery are part of the critical infrastructure of the economy. The restart of our practices and elective surgery as the lockdown ends has made employees anxious with regard to coronavirus exposure in the workplace.^5^ We as surgeons are accustomed to personal protective equipment every day when in the operating room, but office and clinic workers must be compliant with the United States Center for Disease Control guidelines.^6^ The same guiding principles should also be applied to patients and caregivers coming to your clinic or surgical facility.

As we come out of this pandemic, the world will have dramatically changed, and our practices must adapt or perish. We are pleased to be given the opportunity by The Aesthetic Society to start a framework for recovery from the coronavirus pandemic. We additionally hope to create a community feeling for all of us facing this crisis and stressful period of self-isolation and are appreciative of the enormous number of plastic surgeons who logged onto the webinar, the incredible amount of positive feedback.

## Disclosures

The authors declared no potential conflicts of interest with respect to the research, authorship, and publication of this article.

## Funding

The authors received no financial support for the research, authorship, and publication of this article.

## References

[1] Cucinotta D, Vanelli M WHO declares COVID-19 a pandemic. Acta Biomed. 2020;91(1):157-160. 32191675 10.23750/abm.v91i1.9397 PMC7569573

[2] Kernutt Stokes. Top 10 Strategies for maximizing PPP loan forgiveness. Kernutt Stokes website https://kernuttstokes.com/top-10-strategies-for-maximizing-ppp-loan-forgiveness/. Accessed April 22, 2020.

[3] Rao A, Bergen M, Davis S How to fight a price war. Harvard Business Review website. https://hbr.org/2000/03/how-to-fight-a-price-war. Accessed April 22, 2020.

[4] American College of Surgeons. Local resumption of elective surgery guidance. American College of Surgeons website. https://www.facs.org/covid-19/clinical-guidance/resuming-elective-surgery#viewport. Accessed April 22, 2020.

[5] Maloney J, Collas M, Ziobro P Companies improvise reopening plans. Wall St J. 2020;8.

[6] Centers for Disease Control and Prevention. Implementing safety practices for critical infrastructure workers who may have had exposure to a person with suspected or confirmed COVID-19. https://www.cdc.gov/coronavirus/2019-ncov/community/critical-workers/implementing-safety-practices.html. Accessed April 22, 2020.

